# Effects of dietary polyphenol-rich plant products from grape or hop on pro-inflammatory gene expression in the intestine, nutrient digestibility and faecal microbiota of weaned pigs

**DOI:** 10.1186/s12917-014-0196-5

**Published:** 2014-09-04

**Authors:** Anja Fiesel, Denise K Gessner, Erika Most, Klaus Eder

**Affiliations:** Institute of Animal Nutrition and Nutrition Physiology, Justus-Liebig-Universität Giessen, Heinrich-Buff-Ring 26-32, 35392 Giessen, Germany

**Keywords:** Polyphenol, Pig, Intestine, Microbiota

## Abstract

**Background:**

Feeding polyphenol-rich plant products has been shown to increase the gain:feed ratio in growing pigs. The reason for this finding has not yet been elucidated. In order to find the reasons for an increase of the gain:feed ratio, this study investigated the effect of two polyphenol-rich dietary supplements, grape seed and grape marc meal extract (GSGME) or spent hops (SH), on gut morphology, apparent digestibility of nutrients, microbial composition in faeces and the expression of pro-inflammatory genes in the intestine of pigs.

**Results:**

Pigs fed GSGME or SH showed an improved gain:feed ratio in comparison to the control group (*P* < 0.10 for GSGME, *P* < 0.05 for SH). Villus height:crypt depth ratio in duodenum and jejunum as well as apparent total tract digestibility of nutrients were unchanged in the groups receiving GSGME or SH in comparison to the control group. However, the groups receiving GSGME or SH revealed an increased faecal pH value, lower levels of volatile fatty acids and lower counts of *Streptococcus* spp. and *Clostridium* Cluster XIVa in the faecal microbiota (*P* < 0.05). Moreover, both treatment groups had a lower expression of various pro-inflammatory genes in duodenum, ileum and colon than the control group (*P* < 0.05).

**Conclusion:**

The present study suggests that dietary plant products rich in polyphenols are able to improve the gain:feed ratio in growing pigs. It is assumed that an alteration in the microbial composition and anti-inflammatory effects of the polyphenol-rich plant products in the intestine might contribute to this effect.

## Background

Many studies in humans and rodents have shown that dietary polyphenols exert a broad spectrum of beneficial effects with respect to health issues including anti-oxidative or anti-inflammatory properties [1-3]. In contrast, effects of dietary polyphenols on health-related aspects in farm animals have been scarcely investigated so far. In a recent study, we have observed that feeding grape seed and grape marc meal extract (GSGME), a polyphenol-rich by-product of wine or juice processing, improves the gain:feed ratio in weaned pigs [4]. In that study, pigs fed GSGME as a supplement showed also a lower expression of various pro-inflammatory genes and a higher villus height:crypt depth ratio in the duodenum than control pigs. These findings suggest that GSGME as a feed supplement inhibited pro-inflammatory conditions and had a beneficial effect on the absorptive function of the intestine as a result of an increased absorptive surface. However, analyses performed in that study were restricted to duodenum. Thus, it remains unclear whether similar effects of the polyphenol-rich supplement are also occurring in other parts of the digestive tract. Moreover, it is unclear whether these observations are the main reasons for an improvement of feed conversion ratio by feeding GSGME observed in that study. While less is known about the effects of polyphenol-rich plant products in pigs, studies in broilers have shown that plants rich in polyphenols can also influence the microbial composition in a beneficial manner [5]. However, there are also some studies in broilers, mice and rats which exerted adverse effects of polyphenols on nutrient transport in intestinal cells [6-8] and on apparent total tract digestibility of nutrients [8,9]. Thus, the aim of the present study was to investigate the hypothesis that feeding plant products rich in polyphenols not only influences the expression of pro-inflammatory genes and the villus height:crypt depth ratio in the intestine of pigs but might also influences the microbial composition and the expression of nutrient transporters in the intestine and the digestibility of nutrients. It is well known, that the effects of polyphenols on bacterial growth and metabolism vary according to their chemical structure and to their composition in natural products [2]. While a positive effect of GSGME on feed efficiency and health related issues in the small intestine of pigs has been already shown, it is unclear whether plant products with a polyphenol spectrum different from that of grape products exert similar beneficial effects in pigs. In order to address that question, pigs were fed diets supplemented with either grape marc meal extract (GSGME) or spent hops (SH), two inexpensive natural polyphenol-rich sources with a broad, but different, spectrum of native polyphenols.

## Results

### Growth performance of the pigs

There were no differences in average daily gains, daily feed intakes and final body weights between the three groups of pigs (Table 1). However, the SH group showed an improvement of the gain:feed ratio in comparison to the control group (*P* < 0.05, Table 1). In the GSGME group, there was a tendency towards an increased gain:feed ratio in comparison to the control group (*P* ≤ 0.10, Table 1).

**Table 1:** **Growth performance data and apparent total tract nutrient digestibility of crude nutrients in weaned pigs fed a control diet or a diet supplemented with 1% grape seed and grape marc meal extract (GSGME) or 1% spent hops (SH)**

	**Control**	**GSGME**	**SH**
*Growth performance data*			
Initial body weight (kg)	9.8 ± 0.5	10.0 ± 0.5	9.9 ± 0.6
Final body weight (kg)	23.7 ± 2.6	24.1 ± 2.1	23.4 ± 2.0
Daily feed intake (g)^1^	828 ± 115	789 ± 85	762 ± 53
Daily body weight gain (g)	497 ± 63	509 ± 74	497 ± 77
Gain:feed (g/kg)^1^	579 ± 68	620 ± 53^#^	638 ± 83^*^
*Apparent total tract digestibility (%)*			
Crude protein	81.9 ± 3.5	81.0 ± 2.3	78.0 ± 1.4^*^
Crude fiber	55.1 ± 11.8	51.4 ± 7.6	45.1 ± 8.7^*^
Crude fat	65.0 ± 3.4	70.0 ± 3.0^*^	66.2 ± 3.3
N-free extract	90.1 ± 2.4	89.9 ± 2.8	89.4 ± 1.1
Organic matter	80.3 ± 2.5	80.1 ± 2.3	78.7 ± 1.5^#^

Results are shown as mean ± SD (n = 16/group). ^1^Means of two pigs per pen were averaged. ^*^Significantly different from control (*P* < 0.05). ^#^Tended to differ from control (0.05 ≤ *P* ≤ 0.10).

### Nutrient digestibility and relative mRNA abundance of nutrient transporters in duodenum and jejunum

In order to investigate the effect of the polyphenol-rich supplements on nutrient digestibility, apparent total tract digestibilities of crude protein, crude fat, crude fiber and N-free extracts were determined. In the GSGME group, apparent total tract digestibilities of crude protein, crude fiber, N-free extracts and total organic matter were not different from the control group. Apparent total tract digestibility of crude fat increased in comparison to the control group (*P* < 0.05, Table 1). The SH group showed a decrease in the apparent total tract digestibility of crude protein and crude fiber in comparison to the control group (*P* < 0.05, Table 1). The apparent total tract digestibility of organic matter showed a tendency towards a reduction in the SH group compared to the control group (*P* < 0.10, Table 1).

To characterise the effect of the polyphenol-rich supplements on nutrient transporters in the small intestine, relative mRNA abundances of *SLC5A1* (encoding sodium glucose transporter 1; SGLT1), *SLC2A2* and *SLC2A5* (encoding glucose transporter 2 and 5; GLUT2, GLUT5) and *SLC15A1* (encoding intestinal peptide transporter 1; PEPT1) in duodenal and jejunal mucosa were determined. In duodenum, there were no differences in the relative mRNA abundances of the nutrient transporters between the two experimental groups and the control group, with the only exception of a reduced mRNA abundance of *SLC2A5* in the GSGME group (*P* < 0.05, Table 2). In contrast, in jejunum there was a significant down-regulation of various nutrient transporters in in the GSGME and SH groups. In the GSGME group, mRNA abundances of *SLC2A2* (*P* < 0.10), *SLC2A5* and *SLC15A1* (*P* < 0.05) in jejunum were reduced in comparison to the control group (Table 2). In the SH group, mRNA abundances of the glucose transporters considered (*SLC5A1, SLC2A2, SLC2A5*) in jejunum were reduced in comparison to the control group (*P* < 0.05, Table 2).

**Table 2:** **Normalised relative mRNA abundances of nutrient transporters and gut morphology (villus height, crypth depth, villus height:crypt depth ratio) in duodenum and jejunum of weaned pigs fed a control diet or a diet supplemented with 1% grape seed and grape marc meal extract (GSGME) or 1% spent hops (SH)**

	**Control**	**GSGME**	**SH**
*Relative mRNA abundance of nutrient transporters* ^*1*^			
Duodenum			
*SLC5A1*	1.00 ± 0.30	0.81 ± 0.26	0.96 ± 0.19
*SLC2A2*	1.00 ± 0.29	0.93 ± 0.22	1.27 ± 0.34^#^
*SLC2A5*	1.00 ± 0.42	0.47 ± 0.34^*^	0.73 ± 0.27^#^
*SLC15A1*	1.00 ± 0.44	0.97 ± 0.60	1.03 ± 0.56
Jejunum			
*SLC5A1*	1.00 ± 0.38	0.78 ± 0.45	0.62 ± 0.29^*^
*SLC2A2*	1.00 ± 0.49	0.67 ± 0.48^#^	0.54 ± 0.29^*^
*SLC2A5*	1.00 ± 0.63	0.47 ± 0.28^*^	0.46 ± 0.43^*^
*SLC15A1*	1.00 ± 0.51	0.53 ± 0.29^*^	0.69 ± 0.65
*Gut morphology* ^*2*^			
Duodenum			
Villus height (μm)	503 ± 29	520 ± 73	562 ± 81
Crypt depth (μm)	311 ± 51	326 ± 38	354 ± 76
Villus height:crypt depth ratio	1.65 ± 0.27	1.56 ± 0.12	1.58 ± 0.25
Jejunum			
Villus height (μm)	471 ± 94	415 ± 33	425 ± 75
Crypt depth (μm)	277 ± 44	236 ± 42	210 ± 34^*^
Villus height:crypt depth ratio	1.74 ± 0.27	1.86 ± 0.29	1.98 ± 0.33

*SLC2A2* = solute carrier family 2 (facilitated glucose transporter) 2; *SLC2A5* = solute carrier family 2 (facilitated glucose transporter) 5; *SLC5A1* = sodium-glucose transporter 1; *SLC15A1* = solute carrier family 15 (oligopeptide transporter), member 1. Results are shown as mean ± SD (^1^n = 16/group; ^2^n = 6/group) expressed as fold of relative mRNA abundance of the control group. ^*^Significantly different from control (*P* < 0.05). ^#^Tended to differ from control (0.05 ≤ *P* ≤ 0.10).

### Gut morphology

To address potential effects of the polyphenol-rich plant products on absorptive capacity by a changed villus height:crypt depth ratio, histological sections from duodenum and jejunum were analysed as these parts of the small intestine are the main sites of absorption of nutrients and histological changes can be expected in these parts of the small intestine [4]. There was generally less effect of the treatments on gut morphology. While there was no alteration of villus height and crypt depth in both segments of the small intestine in pigs of the GSGME group in comparison to the control group, pigs of the SH group showed a lower crypt depth in jejunum (*P* < 0.05, Table 2). The villus height:crypt depth ratio in both intestinal segments remained unchanged in both experimental groups in comparison to the control group (Table 2).

### Relative mRNA abundances of pro-inflammatory genes in duodenum, ileum and colon

**Figure 1:**
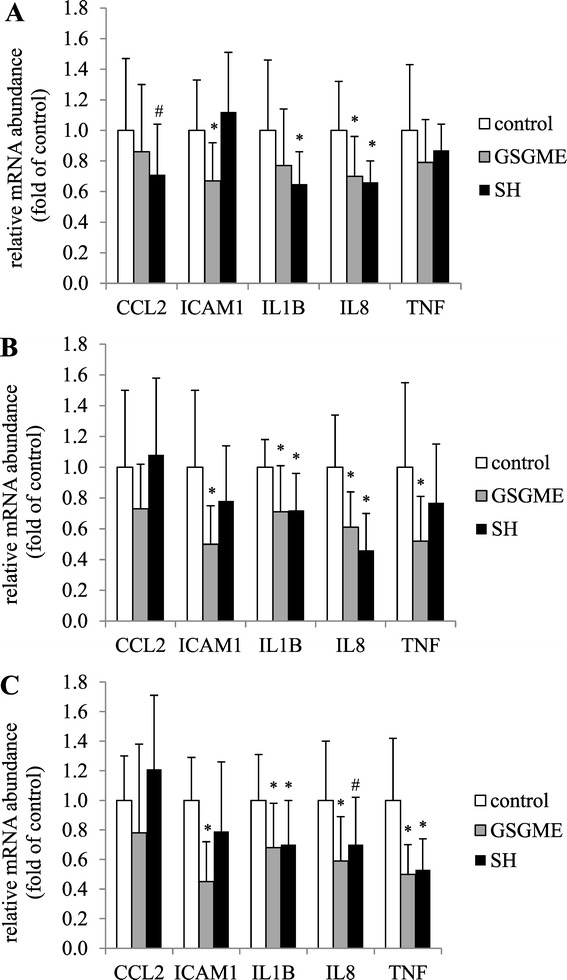
**Relative mRNA abundances of** ***ICAM1, IL1B, IL8, CCL2*** **and** ***TNF*** **in the mucosa of duodenum (A), ileum (B) and colon** ***ascendens*** **C of pigs fed the control diet or diets supplemented with 1% grape seed and grape marc meal extract (GSGME) or 1% spent hops (SH).** Bars represent mean ± SD of 16 pigs per group and are expressed as fold of relative mRNA abundances of the control group. ^*^Significantly different from control (*P* < 0.05). ^#^Tended to differ from control (0.05 ≤ *P* ≤ 0.10). *ICAM1*, intercellular adhesion molecule; *CCL2*, chemokine (C-C motif) ligand 2; *TNF*, tumor necrosis factor; *IL8*, interleukin 8; *IL1B*, interleukin 1 beta.

To investigate the hypothesis that supplementation of polyphenol-rich plant products exert anti-inflammatory effects in the intestine, mRNA abundances of various pro-inflammatory genes (*CCL2, ICAM1, IL1B, IL8, TNF*) in duodenal mucosa were determined. In order to clarify whether active components of the polyphenol-rich plant products are still available and active in in the posterior sections of the intestine, we additionally measured mRNA abundances of these genes in mucosa samples from ileum and colon. Both experimental groups showed significantly lower mRNA abundances of several pro-inflammatory genes in mucosa samples of the three parts of the intestine than the control group. In the GSGME group, there was a reduction of mRNA concentrations of *ICAM1* and *IL8* in duodenum, of *ICAM1, IL1B, IL8* and *TNF* in ileum and of *ICAM1, IL1B, IL8* and *TNF* in colon in comparison to the control group (*P* < 0.05, Figure 1). In the SH group, mRNA concentrations of *IL1B* and *IL8* in duodenum, of *IL1B* and *IL8* in ileum, and of *IL1B* and *TNF* in colon were lower than in the control group (*P* < 0.05, Figure 1).

### Microbial profile and fermentation characteristics

**Figure 2:**
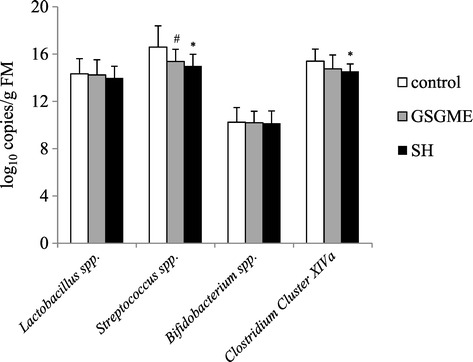
**Occurrence of bacterial groups in faecal samples of pigs fed the control diet or diets supplemented with 1% grape seed and grape marc meal extract (GSGME) or 1% spent hops (SH), determined by qPCR (log**_**10**_ **16S rRNA gene copy number/g fresh matter).** Bars represent mean ± SD of 16 pigs per group. ^*^Significantly different from control (*P* < 0.05). ^#^Tended to differ from control (0.05 ≤ *P* ≤ 0.10).

In order to test the hypothesis that supplementation of polyphenol-rich plant products influences the microbial composition, gene copy numbers of *Lactobacillus* spp., *Streptococcus* spp., *Bifidobacterium* spp. and *Clostridium* Cluster XIVa in faecal samples were determined. Gene copy numbers of *Lactobacillus* spp. and *Bifidobacterium* spp. were not different between the two experimental groups and the control group (Figure 2). However, the GSGME group had a lower number of *Streptococcus* spp. in faecal samples than the control group (*P* < 0.10, Figure 2). The SH group had a lower gene copy number of *Streptococcus* spp. and *Clostridium* Cluster XIVa than the control group (*P* < 0.05, Figure 2).

The GSGME group had a lower concentration of total volatile fatty acids (VFA, *P* < 0.05) in faeces than the control group, due to decreases in the concentrations of acetic (*P* < 0.10), propionic (*P* < 0.05) and valeric acid (*P* < 0.10, Table 3). The SH group showed a tendency towards a reduction of total VFA in faeces compared to the control group (*P* < 0.10, Table 3), due to decreases in the concentrations of propionic (*P* < 0.10), butyric (*P* < 0.05) and valeric acid (*P* < 0.10, Table 3). In line with reduced concentrations of VFA, both experimental groups showed increased faecal pH values in comparison to the control group (*P* < 0.05, Table 3).

**Table 3:** **Concentrations of volatile fatty acids (VFA) and pH value in faeces samples of weaned pigs fed a control diet or a diet supplemented with 1% grape seed and grape marc meal extract (GSGME) or 1% spent hops (SH)**

	**Control**	**GSGME**	**SH**
*VFA (μmol/g digesta)*			
Total VFA	581 ± 111	497 ± 90^*^	524 ± 41^#^
Acetic acid	332 ± 75	282 ± 64^#^	310 ± 31
Propionic acid	141 ± 33	115 ± 21^*^	120 ± 16^#^
Isobutyric acid	10.4 ± 2.5	10.6 ± 2.3	11.8 ± 2.3
Butyric acid	66.1 ± 9.6	61.9 ± 18.5	53.5 ± 13.0^*^
Isovaleric acid	12.9 ± 3.3	13.2 ± 3.9	13.5 ± 2.2
Valeric acid	17.7 ± 4.4	14.2 ± 3.7^#^	14.8 ± 3.8^#^
*Faecal pH value*	5.9 ± 0.1	6.2 ± 0.2^*^	6.2 ± 0.3^*^

Results are shown as mean ± SD (n = 16/group). ^*^Significantly different from control (*P* < 0.05). ^#^Tended to differ from control (0.05 ≤ *P* ≤ 0.10).

## Discussion

Many studies in humans and rodents have shown that dietary polyphenols exert a range of beneficial effects with respect to health issues [2,3,10]. In contrast, potential effects of polyphenols in farm animals on health related aspects have been scarcely investigated so far. In the present study, growing pigs were fed diets supplemented with either GSMGE or SH as dietary supplements rich in polyphenols. GSGME is a by-product of wine and juice processing with gallic acid, catechin, epigallocatechin-3-gallate, epigallocatechin, epicatechin-3-gallate, epicatechin, proanthocyanidins, and anthocyanins as most abundant polyphenols [11]. Hop products are rich in gallic acid, chlorogenic acid, epicatechin, rutin, hyperoside, kaempferol-3-resveratrol, isoquercitrin, xanthohumol and proanthocyanidins [12].

The present study confirms a recent study showing that plant products rich in polyphenols are effective in increasing the gain:feed ratio in growing pigs. In the present study, pigs supplemented with SH exerted a significant improvement of the gain:feed ratio by 10% (*P* < 0.05), while pigs supplemented with GSGME showed a tendency towards an improved gain:feed ratio (+7%, *P* < 0.10). Our recent study in pigs found that supplementation of GSGME causes an increase in the villus height:crypt depth ratio in the duodenum [4]. In agreement with that finding, Viveros *et al.* [5] observed an improved gain:feed ratio and an increased villus height:crypt depth ratio in jejunum in broilers fed a diet supplemented with polyphenol-rich grape pomace extract. Based on the finding of an increased villus height:crypt depth ratio, it was assumed that the plant supplements rich in polyphenols might improve the digestibility of nutrients due to an increased absorptive surface of the intestine. However, in disagreement with our recent pig study and the broiler study of Viveros *et al*. [5], feeding both plant extracts did not influence the villus height:crypt depth ratio in duodenum or jejunum in this study. Interestingly, another study in weaned pigs observed that feeding polyphenol-rich red-wine pomace exerts even an inhibitory effect on jejunal villi growth [13]. These findings suggest that polyphenol-rich plant products do not have a consistent effect on the gut morphology. It rather seems that the effects of polyphenol-rich plant products on villi heights and crypt depths depend on various factors, probably including the concentrations of diverse polyphenols. In addition, the plant products did not improve the apparent total tract digestibility of the crude nutrients and organic matter. Indeed, the digestibilities of crude protein and crude fiber were even slightly reduced in pigs fed SH as a supplement. The finding of a decreased apparent digestibility of protein is in agreement with a recent study in broilers which found a decreased apparent ileal digestibility of protein after feeding grape seed extracts [6]. In overall, these findings indicate that the improved gain:feed ratio by feeding either GSGME or SH was not due to alterations in gut morphology (villus height:crypt depth ratio) or an increased digestibility of nutrients from the diet.

Several studies have shown that dietary polyphenols are able to influence nutrient uptake into intestinal cells. For instance, flavonoid glycosides and non-glycosylated polyphenols including epigallocatechingallate, epigallocatechin or epicatechingallate have been shown to decrease glucose uptake in Caco-2 cells [14]. Other studies observed an interaction of polyphenols with SGLT1 [15,16] or GLUT2 [17] in a non-competitive manner, associated with a reduced intestinal uptake of glucose. Based on these findings, it has been suggested that polyphenols might act as potent inhibitors of glucose absorption, and thus might be promising agents in the treatment of obesity [17]. Indeed, human intervention studies showed a reduction in glycemic index after ingestion of red wine, sugar cane extract, coffee, berries or apple juice, indicating that polyphenols present in these foods might have slowed down the absorption of glucose [18]. In the present study, we observed that feeding GSGME and SH leads to a strong down-regulation of SGLT1, GLUT2, GLUT5 and PEPT1 at the transcriptional level in the jejunum. SGLT1 is considered as the apical intestinal transporter responsible for the majority of luminal glucose transport across intestinal epithelium [19]. GLUT2, which is located at the basolateral membrane of the enterocyte, is a facilitative transporter for glucose and fructose which operates with low affinity and high capacity [20]. GLUT5 is a low affinity and high capacity transporter specific for fructose, located at both, the apical and the basolateral site of the enterocyte [20]. PEPT1 is an apical electrogenic proton/peptide symporter which is responsible for the absorption of the majority of amino acids as di- or tripeptides [21]. We are not aware of any other study dealing with the effects of polyphenols on the expression of nutrient transporters in intestinal cells. However, our study suggests that a reduced expression of the nutrient transporters involved in transport of glucose could contribute to a reduction of the glycemic index observed in humans after ingestion of sources rich in polyphenols [18]. In a similar manner, a reduced expression of PEPT1 in pigs fed polyphenol-rich plant products could cause a reduction of the absorption of di- and tripeptides. Irrespective of this, we observed that the apparent total tract digestibility of N-free extracts, the fraction consisting mainly of starch, was not diminished by supplementation of the polyphenol-rich plant products. This suggests that the reduction of glucose transporters was uncritical with respect to total tract digestibility of glucose. On the other hand, a down-regulation of PEPT1 could have contributed to the slightly decreased apparent digestibility of dietary protein observed in the pigs fed the diet supplemented with SH. However, we are aware that determination of total tract digestibility is confounded by losses of nutrients due to microbial activity in colon. Thus, determination of praecaecal digestibility of nutrients would provide a more reliable picture of the effect of polyphenols on the digestibility of nutrients in the small intestine.

Previous studies in rats and broilers have shown that polyphenols are able to cause a shift in the microbial population in the intestinal tract [5,22,23]. In a rat model of colon cancer administration of polyphenols from red wine caused a decrease of *Propionibacteria, Bacteroides* and *Clostridia* and an increase of *Lactobacilli* and *Bifidobacteria* in colonic content [23]. In a study with broilers feeding grape pomace extract or grape seed extract increased counts of beneficial ileal bacteria populations such as *Enterococcus* and decreased counts of potential pathogens such as *Clostridium* were observed [5]. Moreover, *in vitro* studies demonstrated antibacterial activities of polyphenols from grape seed extract or phenolic compounds from different wines against different bacteria, including *Enterococcus faecalis*, *Escherichia coli* and *Streptococcus enteritidis* [24-26]. In agreement with those findings, we observed for the first time that plant products rich in polyphenols may be able to influence the microbial population in the intestine of pigs. Our analyses in faecal samples showed a reduction of *Streptococcus* spp. and *Clostridium* Cluster XIVa in pigs fed polyphenol-rich plant products, a finding which is similar with that of the broiler study of Viveros *et al*. [5]. While *Lactobacilli* and *Bifidobacteria* are considered beneficial for intestinal function [27,28], *Clostridia* have detrimental effects in intestinal mucosa [29,30]. The findings of a reduced concentration of total volatile fatty acids and an increased pH value in faecal samples of pigs fed the plant products rich in polyphenols indicate that there was generally a reduced microbial fermentation in these pigs which confirms the view that polyphenols could have an antimicrobial effect.

In agreement with our recent study [4], we observed that feeding polyphenol-rich plant products cause a down-regulation of various pro-inflammatory cytokines, including *ICAM1, IL1B, IL8* and *TNF* in the mucosa of various segments of the intestine. Noteworthy, these genes are regulated by nuclear factor κB (NF-κB), the master regulator of inflammation [31-33]. Several *in vitro* or *in vivo* studies, mainly performed in rodent models of acute or chronic colitis have already shown that dietary polyphenols are able to act anti-inflammatory by inhibiting transactivation of NF-κB [1,4,34,35]. Although a direct inhibitory effect of polyphenols on the activity of NF-κB has been well established, it is possible that the anti-inflammatory effects observed in this study could be - at least in part - due to antimicrobial effects of the polyphenol-rich plant products. The finding that anti-inflammatory effects were observed not only in duodenum but also in ileum and colon indicates that the active components of the polyphenol-rich plant products were not completely absorbed or degraded in the anterior part of the small intestine but were at least in part available and active in the posterior parts of the intestine.

It has been shown that mucosa-associated bacteria can trigger pro-inflammatory gene transcription by invading epithelial cells, interacting with specific receptors (e.g. toll-like receptors) or through direct inhibition or activation of NF-κB transcriptional activity [36-38]. Irrespective of the exact mode of action NF-κB inhibition, the present study confirms the concept that dietary polyphenols might provide a useful dietary strategy to inhibit inflammation in the gut of pigs.

One aim of this study was to compare the effects of plant products which vary in their spectrum of polyphenols. We found that the effects on the parameters considered in this study, including gain:feed ratio, digestibility of nutrients, effects on nutrient transporters as well as microbial composition and production of volatile fatty acids - were largely similar for both plant sources of polyphenols. From this finding we conclude that the effects observed were probably not induced by few individual polyphenols but by a greater range of different polyphenols.

In overall, the present study confirms that feeding polyphenol-rich plant products improves the gain:feed ratio in growing pigs. It has become apparent that this effect was not induced by effects on gut morphology (villus height:crypt depth ratio) or digestibility of nutrients. More likely, alterations of the microbial composition as well as anti-inflammatory properties might contribute to the beneficial effects on the gain:feed ratio. Moreover, it is possible that dietary polyphenols exert beneficial effects in intermediary metabolism which could contribute to an increased efficiency of nutrients for animal growth.

## Conclusions

In conclusion, the present study confirms that supplementation of GSGME or SH, two polyphenol-rich plant products, improves the gain:feed ratio in growing pigs. As gut morphology (villus height:crypt depth ratio) and apparent total tract digestibility of nutrients were in overall not influenced, the improvement of the gain:feed ratio by feeding the plant products was probably not due to enhanced nutrient digestibility. It is shown that feeding GSGME or SH causes an alteration in the microbial composition, with a decrease of *Streptococcus* spp. and *Clostridium* Cluster XIVa, and a down-regulation of several pro-inflammatory genes in the mucosa of various parts of the intestine. It is assumed that these effects may contribute to the increased gain:feed ratio observed in the pigs fed the polyphenol-rich plant products.

## Methods

### Animals and diets

The feeding trail was performed with forty-eight five week old crossbreed pigs [Piétrain x (German Landrace × German Edelschwein)], which were randomly assigned to three groups of 16 pigs each and housed in pairs in flat-deck in a room with controlled temperature (23 ± 2°C), relative humidity (50-60%), and light from 06.00 to 19.00. The pigs were fed two nutritionally adequate basal diets in phases I (<15 kg body weight) and II (>15 kg body weight) which were composed to meet the recommendations of the German Society for Nutrition Physiology (GfE) for growing pigs in the respective body weight ranges [39]. Composition and nutrient concentration of these diets are shown in Table 4. The first group (control group) received the basal diet without any supplement. The second group (GSGME group) received the basal diets supplemented with 1% GSGME (Anta®Ox E, Dr. Eckel GmbH, Niederzissen, Germany) in exchange for 1% wheat. Crude nutrient concentrations of GSGME were (in g/kg): crude fiber (346), crude protein (110), crude fat (41), crude ash (29). The total polyphenol content was 8.5% according to the manufacturers' specifications. The third group (SH group) received the basal diet supplemented with 1% SH (Anta®Phyt H, Dr. Eckel GmbH, Niederzissen, Germany) in exchange for 1% wheat. Crude nutrient concentrations of SH were (in g/kg): crude fiber (218), crude protein (212), crude fat (13), crude ash (86). The total polyphenol content was around 5% according to the manufacturers' specifications. The diets were offered for free access. Unconsumed feed was weighed daily. Water was also provided *ad libitum* from a nipple drinker system. The pigs were weighed once per week. All experimental procedures were in strict accordance with the recommendations in the guidelines for the care and use of laboratory animals [40] and the Appendix A of European Convention for the Protection of Vertebrate Animals used for Experimental and other Scientific Purposes [41]. In accordance with article 4 par. 3 of the German Animal Welfare Law [42] all animals were humanely killed for scientific purpose approved by the Animal Welfare Officer of the Justus-Liebig-University, JLU No. 439_M.

**Table 4:** **Composition of the basal experimental diets fed in phase I (body weight < 15 kg) and II (body weight > 15 kg)**

	**Phase I**	**Phase II**
*Composition (g/kg)*		
Wheat	381.7	406.4
Barley	315	302
Soy bean meal (44% crude protein)	250	240
Soy oil	15	15
Mineral and vitamin premix*	33.5	33.4
L-Lysine	2.6	1.5
DL-methionine	1.0	0.5
L-threonine	1.2	0.7
Titanium dioxide	-	0.5
*Concentration of nutrients*		
Metabolizable energy (MJ/kg)^†^	13.7	13.3
Dry matter (%)^‡^	88.8	88.2
Crude protein (%)^‡^	19.8	18.5
Crude fiber (%)^‡^	3.3	4.3
Crude fat (%)^‡^	3.5	4.0
Crude ash (%)^‡^	5.1	5.0
Digestible lysine (%)^¥^	1.16	1.05
Digestible methionine + cysteine (%)^¥^	0.62	0.57
Digestible threonine (%)^¥^	0.69	0.63
Digestible tryptophan (%)^¥^	0.21	0.21

*The mineral and vitamin premix (Bergin Novamast, Bergophor, Kulmbach, Germany) provided the following per kg diet: 1.34 g lysine, 1,020 FYT phytase, 102 mg iron, 102 mg manganese, 102 mg zinc, 20.4 mg copper, 2.21 mg iodine, 0.44 mg selenium, 13,400 IE vitamin A, 2,244 IE vitamin D_3_, 102 mg vitamin E, 2.55 mg vitamin K, 2.55 mg vitamin B_1_, 6.8 mg vitamin B_2_, 5.1 mg vitamin B_6_, 34 μg vitamin B12, 34 mg nicotinic acid, 17 mg Ca-D-pantothenic acid, 1 mg folic acid, 136 μg biotin, 340 g choline chloride.

^†^Calculated according to recommendations of German Society for Nutrition Physiology.

^‡^Analysed (mean values of three analyses per diet).

^¥^calculated using tabular values from AMINODat® 4.0 (Evonik Industries AG, Essen, Germany).

### Sample collection

After 4 weeks of feeding, the pigs were anesthetised and exsanguinated for sample collection. Blood samples were collected into EDTA polyethylene tubes (Sarstedt, Nürnbrecht, Germany) and plasma was separated by centrifugation (1100 g, 10 min) at 4°C. Plasma samples were stored at -20°C. The gastrointestinal tract was removed and duodenum, jejunum (medial part), ileum and colon *ascendens* (proximal part of *gyri centripetales*) were sampled. Mucosal samples of all picked segments were taken by scraping off the mucosa with a cell lifter (Corning Incorporated, Corning, USA) after removing and flushing with 0.9% NaCl (w/v). Samples were snap-frozen in liquid nitrogen and stored at -80°C pending analysis. For histological analysis, tissue samples of duodenum and jejunum (medial part) were washed three times in 0.9% NaCl (w/v) and PBS and stored for 24 hours in 4% paraformaldehyde (Merck, Darmstadt, Germany) at 4°C. Faecal samples were removed from the rectum rapidly after slaughtering. pH value was measured with a pH meter (pH-Meter 646, Knick, Berlin, Germany) and the samples were stored at -20°C.

### Nutrient digestibility

Feed and faecal samples were stored at -20°C until digestibility was determined using titanium dioxide (TiO_2_) as an indigestible marker. The samples were analysed for dry matter (DM), crude ash (CA), crude protein (CP), crude fat (CL), crude fiber (CF) and TiO_2_. DM, CA, CL and CF were analysed according to the official German VDLUFA methodology [43]. CP (N × 6.25) was determined by CN-Analysator (vario MAX N/CN, elementar Analysesysteme GmbH, Hanau, Germany). The concentrations of TiO_2_ in diets and faecal samples were measured photometrically by the method of Brandt and Allam [44]. Apparent total tract digestibility coefficients of organic matter and crude nutrients were calculated with following formula:

$$ \begin{array}{l}\mathrm{Apparent}\ \mathrm{total}\ \mathrm{tract}\ \mathrm{digestibility}\ \left(\%\right) = \\ {}100\ \hbox{--}\ \left\{\left(\%\ {\mathrm{TiO}}_2\mathrm{in}\ \mathrm{diet}/\%\ {\mathrm{TiO}}_2\mathrm{in}\ \mathrm{faeces}\right) \times \left(\%\ \mathrm{nutrient}\ \mathrm{in}\ \mathrm{faeces}/\%\ \mathrm{nutrient}\ \mathrm{in}\ \mathrm{diet}\right)\times 100\right\}\end{array} $$

### Total RNA isolation, cDNA synthesis and quantitative real-time polymerase chain reaction (qPCR) analysis

For qPCR analysis, all removed parts of the intestine (duodenum, jejunum, ileum, colon *ascendens*) were selected. RNA isolation, cDNA synthesis and qPCR analysis were performed as described recently in detail [45]. In brief, total RNA was extracted from 20 mg mucosa aliquots using TRIzol reagent (Invitrogen, Karlsruhe, Germany) according to the manufacturer’s protocol. Purity and concentration of total RNA were estimated from the optical density at 260 and 280 nm (Infinite 200 M microplate reader, Tecan, Männedorf, Switzerland). RNA integrity was confirmed by visualisation of 18S and 28S rRNA bands by formaldehyde-agarose gel electrophoresis. qPCR analysis was performed using KAPA SYBR FAST qPCR Universal Mastermix (Peqlab, Erlangen, Germany) and gene-specific primer pairs from Eurofins MWG Operon (Ebersberg, Germany) in a Rotor-Gene 2000 system (Corbett Research, Mortlake, NSW, Australia). Gene-specific primer pairs are listed in Table 5. Expression values were normalised using GeNorm normalisation factor according to Vandesompele *et al.* [46]. The normalisation factor was calculated as the geometric mean of expression data of the three most stable out of six tested potential reference genes. Means and SD were calculated from normalised expression data for samples of the same treatment group. The mean of the control group was set to 1 and mean and standard deviation (SD) of the GSGME or SH group were scaled proportionally.

**Table 5:** **Characteristics of primers used for qPCR analysis**

**Gene** ^**1**^	**Forward primer (from 5′to 3′)** **Reverse primer (from 5′to 3′)**	**PCR product size (bp)**	**NCBI GenBank**
Reference genes			
*ATP5G1*	CAGTCACCTTGAGCCGGGCGA	94	NM_001025218.2
	TAGCGCCCCGGTGGTTTGC		
*ACTB*	GACATCCGCAAGGACCTCTA	205	XM_003124280.3
	ACATCTGCTGGAAGGTGGAC		
*GAPDH*	AGGGGCTCTCCAGAACATCATCC	446	NM_001206359.1
	TCGCGTGCTCTTGCTGGGGTTGG		
*GPI*	CACGAGCACCGCTCTGACCT	365	NM_214330.1
	CCACTCCGGACACGCTTGCA		
*RPS9*	GTCGCAAGACTTATGTGACC	327	XM_005664825.1
	AGCTTAAAGACCTGGGTCTG		
*SDHA*	CTACGCCCCCGTCGCAAAGG	380	DQ402993
	AGTTTGCCCCCAGGCGGTTG		
NF-κB and nutrient transporter target genes			
*CCL2*	GGTCCTTGCCCAGCCAGATGC	170	NM_214214.1
	CTGCACAGATCTCCTTGCCCGC		
*ICAM1*	CGGTGGCAGCCGTGGCTATC	208	NM_213816.1
	TTGATGCAGCCCCGCTCGTC		
*IL1B*	GTTCTCTGAGAAATGGGAGC	143	NM_214055.1
	CTGGTCATCATCACAGAAGG		
*IL8*	ACTTCCAAACTGGCTGTTGC	120	NM_213867.1
	GGAATGCGTATTTATGCACTGG		
*SLC2A2*	GCTGGATGGGGAAGCCAAAGCA	355	NM_001097417.1
	AGAGCGTCGCCCTGCCTTCT		
*SLC2A5*	CTGACACTGGTGCTTGCTTT	156	EU012359.2
	TTCGCTCATGTATTCCCCGA		
*SLC5A1*	GTGGCGGACAGTAGTGAACA	89	NM_001164021.1
	AGAAGGCAGGATTTCAGGCA		
*SLC15A1*	CAGACTTCGACCACAACGGA	99	NM_214347.1
	TTATCCCGCCAGTACCCAGA		
*TNF*	CATGAGCACTGAGAGCATGA	170	NM_214022.1
	CGATAACTTCGAAGTGCAGT		
Bacterial group			
*Bifidobacterium* spp.	TCGCGTC(A/T)GGTGTGAAAG	243	
	CCACATCCAGC(A/G)TCCAC		
*Clostridium* Cluster XIVa	AAATGACGGTACCTGACTAA	485	
	CTTTGAGTTTCATTCTTGCGAA		
*Lactobacillus* spp.	AGCAGTAGGGAATCTTCCA	341	
	CACCGCTACACATGGAG		
*Streptococcus* spp.	AGAGTTTGATCCTCCGTCAG	144	
	GTTAGCCGTCCCTTTCTGG		

^1^ *ATP5G1* = ATP synthase lipid-binding protein; *ACTB* = actin beta; *GAPDH* = glyceraldehyde 3-phosphate dehydrogenase; *GPI* = glucose-6-phosphate isomerase; *RPS9* = ribosomal protein; *SDHA* = succinate dehydrogenase complex, subunit A; *CCL2* = chemokine (C-C motif) ligand 2; *SLC2A2* = solute carrier family 2 (facilitated glucose transporter) 2; *SLC2A5* = solute carrier family 2 (facilitated glucose transporter) 5; *SLC5A1* = sodium-glucose transporter 1; *SLC15A1* = solute carrier family 15 (oligopeptide transporter), member 1; *ICAM1* = intercellular adhesion molecule; *IL* = interleukin; *TNF* = tumor necrosis factor.

### Cryosectioning for light microscopy

Tissue samples of duodenum and jejunum were removed and fixed in 4% paraformaldehyde (MERCK, Darmstadt, Germany). After 24 hours, samples were washed three times with 1× PBS followed by incubation in a cryoprotectant 1× PBS solution containing 30% sucrose until embedding the samples in Tissue-Tek (Hartenstain, Würzburg, Germany) and cryosectioning on a cryostat microtome (Microme HM 500, MICROM international GmbH, Walldorf, Germany) to 20 μm thickness. The unstained sections were analysed by light microscopy (Leica DMI 6000B) at 100× magnification for measuring villus height and crypt depth and calculating the ratio of villus height to crypt depth, which were reported as mean length of 15 well oriented and representative villi and crypts from 6 pigs per group.

### DNA extraction and qPCR analysis of faecal bacteria composition

Total bacterial DNA was extracted from 30 mg lyophilised faeces with the Repeated Bead Beating Plus Column Method [47]. This protocol includes two steps of bead beating, which were done by TissueLyser (Qiagen, Hilden, Germany) and 0.1 mm zirconium-silica beads (Biospec Products, Bartlesville, USA). Using the QIAamp DNA Stool Mini Kit columns (Qiagen, Hilden, Germany) according to the manufacturer’s protocol, the DNA was isolated by sequential precipitations and finally purified. Purity of total DNA was estimated from the optical density at 230, 260 and 280 nm (Infinite 200 M microplate reader, Tecan, Männedorf, Switzerland). A 260/280 value of ~ 1.8 is generally accepted as pure DNA. The 260/230 ratio is also an indicator of contamination. The expected 260/230 values are commonly in the range of 2.0 - 2.2. For the analysed faecal samples the observed 260/280 ratio was 1.82 ± 0.03, and the 260/230 ratio 2.07 ± 0.18.

Quantitative PCR analysis was performed as described above on all faecal DNA extracts using group-specific primers targeting the 16S rRNA gene of *Lactobacillus* spp., *Bifidobacterium* spp., *Streptococcus* spp. and *Clostridium* Cluster XIVa which are listed in Table 5. Standard curves were generated using serial dilutions of the purified and quantified PCR products.

### Faecal volatile fatty acid composition

The faecal volatile fatty acid composition was analysed by gas chromatography. 100 mg aliquots of the faecal samples were mixed with 1 ml internal standard working solution consisting of 0.03 g crotonic acid and 5 ml o-phosporic acid in 100 ml [48]. The mixture was extracted by vortexing for 1 min and centrifugation at 20,000 g at 4°C for 10 min. 1 μl of the extract was injected into a gas chromatograph (Clarus 580 GC system, Perkin Elmer, Waltham, USA) equipped with a flame-ionisation detector and a split injector. Fatty acids were separated on a polar capillary column (10 m free fatty acid phase, 0.32 mm internal diameter, 0.25 μm film thickness; Macherey and Nagel, Düren, Germany). Individual fatty acids were identified by comparing their retention times with those of individually purified standards. The fatty acid concentrations were calculated from the peak areas relative to the peak area of crotonic acid as the internal standard.

### Statistical analysis

Data were statistically evaluated by one-way ANOVA using the Minitab Statistical Software (Rel. 13.0, State College, PA, USA). The Student’s *t* test was used for the comparison of two groups (control vs. GSGME or control vs. SH). Means were considered significantly different for *P* < 0.05 and tended to differ when 0.05 ≤ *P* ≤ 0.10. Data in the text are presented as mean ± SD.
